# Oxidative Stress and Cardiovascular-Renal Damage in Fabry Disease: Is There Room for a Pathophysiological Involvement?

**DOI:** 10.3390/jcm7110409

**Published:** 2018-11-02

**Authors:** Verdiana Ravarotto, Francesca Simioni, Gianni Carraro, Giovanni Bertoldi, Elisa Pagnin, Lorenzo A. Calò

**Affiliations:** Department of Medicine, Nephrology, Dialysis and Transplantation Unit, University of Padova, 35128 Padova, Italy; verdiana.ravarotto@gmail.com (V.R.); francesca.simioni@gmail.com (F.S.); carraro.gianni@gmail.com (G.C.); giovanni.bertoldi92@gmail.com (G.B.); elisa.pagnin@unipd.it (E.P.)

**Keywords:** Fabry disease, Fabry disease cardiovascular-renal damage, oxidative stress, pathophysiology

## Abstract

Fabry disease is an X-linked lysosomal storage disease caused by mutations in the GLA gene that lead to a reduction or an absence of the enzyme α-galactosidase A, resulting in the progressive and multisystemic accumulation of globotriaosylceramide. Clinical manifestation varies from mild to severe, depending on the phenotype. The main clinical manifestations are cutaneous (angiokeratomas), neurological (acroparesthesias), gastrointestinal (nausea, diarrhea abdominal pain), renal (proteinuria and kidney failure), cardiovascular (cardiomyopathy and arrhythmias), and cerebrovascular (stroke). A diagnosis of Fabry disease can be made with an enzymatic assay showing absent or reduced α-galactosidase A in male patients, while in heterozygous female patients, molecular genetic testing is needed. Enzyme replacement therapy (ERT) with recombinant human α-galactosidase is nowadays the most-used disease-specific therapeutic option. Despite ERT, cardiocerebrovascular-renal irreversible organ injury occurs, therefore additional knowledge and a deeper understanding of further pathophysiological mechanisms leading to end organ damage in Fabry disease are needed. Recent data point toward oxidative stress, oxidative stress signaling, and inflammation as some such mechanisms. In this short review, the current knowledge on the involvement of oxidative stress in cardiovascular-renal remodeling is summarized and related to the most recent evidence of oxidative stress activation in Fabry disease, and clearly points toward the involvement of oxidative stress in the pathophysiology of the medium- to long-term cardiovascular-renal damage of Fabry disease.

## 1. Introduction

Fabry disease (FD, OMIM 301500) is an X-linked lysosomal storage disorder caused by the deficient activity of the enzyme alfa-galactosidase A (α-Gal A). The reduced or absent activity of this lysosomal enzyme, whose task is to degrade the cellular membrane of glycosphingolipids, leads to the progressive accumulation of glycolipids—primarily globotriaosylceramide (Gb_3_ or GL-3) and its deacylated form, globotriaosylsphingosine (lyso-GL-3)—within lysosomes, which are ubiquitous cellular organelles. Gb_3_ accumulates in a wide range of cells including vascular endothelial cells, podocytes, cardiomyocytes, arterial smooth muscle cells, nerve cells, and other cell types, resulting in a multisystemic disorder [1]. Without treatment with the most-used disease-specific therapeutic option, enzyme replacement therapy (ERT) with recombinant human α-galactosidase, the deposits within the kidney, heart, and cerebrovascular system can lead to prominent complications such as end stage renal disease or cardiovascular events, which in turn can be responsible for premature death [2,3]. Based on this progression, early detection of the disease is fundamental in order to start the appropriate treatment.

However, no clear evidence has emerged that ERT is able to alter the natural course of Fabry disease-associated cardiac and cerebrovascular diseases and nephropathy, especially in the late diagnosis of Fabry disease. This suggests that these adverse outcomes do not entirely depend on the simple direct effect of glycosphingolipids accumulation [4].

In the close relationship between kidney damage and cardiovascular disease, traditional (diabetes, hypertension, obesity, smoking) and non-traditional (prothrombotic and proinflammatory states, endothelial dysfunction, oxidative stress) risk factors play a very important role. Both traditional and non-traditional risk factors have a strong impact on the life expectancy of a patient with chronic kidney disease (CKD) [5]. A vicious circle best describes the relationship of CKD with the development of hypertension, cardiovascular-renal remodeling, and cardiovascular adverse events, including cerebrovascular events [6]. Renal disease and cardiovascular disease (CVD) are, in fact, associated with endothelial dysfunction, inflammation, and oxidative stress, which have similar roles in both CVD and CKD via interconnections between oxidative stress, inflammation, and endothelial dysfunction. Through these interconnections, these entities coexist and communicate with each other, thereby exacerbating the processes underpinning these different entities with the end result of high morbidity and mortality in these high-risk patients [6]. The exact sequence of events is unclear, but certainly involves the induction of oxidative stress with increased vascular superoxide and free radical formation, which then lead to endothelial dysfunction. CKD and dialysis patients exhibit increased plasma levels of oxidative stress-related proteins, which reduce the availability of nitric oxide (NO) and produce endothelial dysfunction [6]. In particular, the increased prooxidant activity is due to the excessive production of reactive oxygen species (ROS), which, interacting with NO via a chemical reaction, causes reduced NO availability and thereby the reduction of its vasodilatory, anti-inflammatory, and antioxidant effects along with a resultant reduction in antioxidant defenses. In addition to the relationship between CKD and oxidative stress, an association of oxidative stress with myocardial remodeling has been shown by the contrasting myocardial remodeling in vivo and in vitro, as revealed by study results in hypertensive patients compared to Bartter’s and Gitelman’s syndrome patients, which are considered to be the mirror image of hypertensive patients [7].

In this paper, the current knowledge on the involvement of oxidative stress in cardiovascular-renal remodeling is summarized and related to the most recent evidence, clearly pointing toward the involvement of oxidative stress in the pathophysiology of the medium- to long-term cardiovascular-renal damage of Fabry disease.

## 2. Oxidative Stress, Inflammation, Renal, and Cardiovascular Damage

Oxidative stress represents an imbalance between prooxidant and antioxidant factors, in favor of the former, which leads to potential damage and is a key player in processes such as ageing, atherosclerosis, and acute inflammatory disease [8]. Oxidative stress is mediated in part by ROS, which are normally produced in controlled amounts by endogenous defense mechanisms. When the control of ROS homeostasis is lost, resulting in non-physiological ROS concentrations, this overwhelms the recovery capacity of the cells and causes damage to proteins, membrane lipids, and nucleic acids [9]. Oxidative stress, inflammation, and endothelial dysfunction are detectable, even at the very early stages of CKD. The increased levels of circulating ROS, IL-1, and the lower bioavailability of NO promote dysfunctional vascular smooth muscle cells to migrate towards intima to cause intimal hyperplasia and the deposition of abnormal extracellular matrix and hyaline material. This situation leads to vascular calcification, with the stiffening of arteries and high pulse pressure [10,11], which become evident in CKD. In addition, these mediators have been reported to play an essential role in the epithelial cells of renal tubules which can undergo a process called epithelial-to-mesenchymal transition (EMT) [12,13].

In CKD and dialysis patients, the involvement of oxidative stress and oxidative stress-related proteins has been widely demonstrated, as has the beneficial effect of oxidative stress inhibition with green tea flavonoids, which significantly reduce the expression of oxidative stress-related protein [14]. The prevention of oxidative stress has a strong impact not only on the production of proteins of rapid response to injury, but also on the reduction of those involved in fibrosis and CV remodeling, such as the extracellular signal-regulated kinases (ERK) 1/2, which are the direct oxidative stress effector proteins for CV remodeling and atherosclerosis [15]. The phosphorylation of ERK 1/2 was found to be significantly reduced after the treatment of hemodialysis patients with green tea [14].

Oxidative stress has a marked influence on cardiovascular remodeling. Left ventricular hypertrophy (LVH) was shown to be correlated with the oxidation of low density lipoproteins (LDL) and p22^phox^ expression [14], a subunit of NADPH oxidases that is crucial for the production of superoxide [16]. The nutraceutical treatment with green tea significantly reduced the cardiac mass alongside the oxidation of LDL in dialysis patients, emphasizing the correlation between oxidative stress effects on lipids, which inhibits macrophage mobility favoring their accumulation, and the formation of the initial stages of atherogenic processes and myocardial fibrosis in CKD [14,17].

Another pathway deeply involved in the fibrotic responses both in kidney and cardiovascular systems via the induction of oxidative stress is the RhoA/Rho kinase pathway, which also influences the upregulation of ROS via the induction of NADPH oxidases [7,18,19,20]

Recently it has been reported that Rho kinase activity, the effector of the monomeric G protein RhoA, is higher in hypertensive patients with LVH compared to hypertensive patients without LVH [21]. It plays a significant role in both the regulation of blood pressure and the induction of cardiovascular-renal remodeling, and has also been proposed as an LVH marker [21,22]. The Rho kinase pathway is triggered by upstream molecules such as Ang II, ET-1, norepinephrine, cytokines, and growth factors. Once their receptor is activated, the intracellular signaling for Rho kinase involves specific mediators such as p63RhoGEF, which stimulates the RhoA and, in turn, activates Rho kinase [23,24]. The downstream target of Rho kinase is an inhibitory subunit of the myosin light chain kinase, the myosin light chain phosphatase subunit target (MYPT)-1, which is inactivated via a Rho kinase-induced inhibiting phosphorylation. MYPT-1, when phosphorylated by Rho kinase, has suppressed phosphatase activity and therefore prolongs the myosin light chain kinase effects of smooth muscle contraction and allows proliferative-profibrotic signals such as those on NADPH oxidase activation, oxidative stress, and NO suppression [25].

In the long term, Rho kinase activation leads to cardiac hypertrophy, remodeling, and ventricular dysfunction. On the contrary, the inhibition of the RhoA-Rho kinase pathway has been suggested to lead to cardiovascular-renal protection by in vitro and in vivo studies in rats with Ang II-induced LVH and cardiomyocyte hypertrophy [20]. The downregulation of Rho kinase signaling is associated with the upregulation of the nitric oxide system and increased nitric-oxide-mediated vasodilation. Furthermore, cardiovascular-renal protection is clearly shown in models of endogenous Ang II signaling antagonism such as Bartter’s and Gitelman’s syndromes [7]. Finally, Rho kinase has been shown to be activated not only in dialysis and CKD patients; it was found to be higher in those patients who had LVH compared to those without LVH. In addition, the Rho kinase inhibitor fasudil showed a dose-dependent reduction of Rho kinase activity in these CKD and dialysis patients [22].

## 3. Oxidative Stress and Fabry Disease

As reported above, no clear evidence has emerged, particularly in the late diagnosis of Fabry disease, that ERT is able to alter the natural course of Fabry disease-associated cardiac or cerebrovascular disease or nephropathy. This suggests that these adverse outcomes do not entirely depend on the simple direct effect of glycosphingolipids accumulation [4]. In patients with a high risk of cardiovascular disease, cardiovascular-renal remodeling represents the most common cause of excess morbidity and mortality. Oxidative stress plays a central role in the onset and progression of atherosclerosis, inflammatory diseases, and cardiovascular-renal remodeling, starting with the induction of endothelial dysfunction [6].

In Fabry disease, the presence of oxidative stress has been shown in terms of elevated plasma levels of proinflammatory cytokines and some markers of oxidative stress. There is, in fact, evidence showing that oxidative stress may be involved in the pathophysiology of Fabry disease. Ascorbate, a potent antioxidant, was, in fact, found to decrease hypoperfusion in Fabry patients under ERT treatment [26]. Dermal vascular nitrotyrosine in excess, a marker of oxidative stress, was found in biopsies from Fabry patients [27]. In addition, Gb3 has been shown to increase ROS generation and the expression of adhesion molecules in cultured Fabry endothelial cells, and plasma from patients treated with ERT induced increased ROS generation in cultured endothelial cells [28]. Finally, in apolipoprotein-E-deficient mice, α galactosidase A deficiency accelerated atherosclerosis and caused increased nitrotyrosine in their plaque [29].

Gb3 accumulation, a hallmark of Fabry disease, is not limited to the lysosomes—it is also found to be increased in the plasma membranes and caveolae of endothelial cells. It has recently been suggested that Gb3 accumulation in the endothelium is able to dysregulate the activity of endothelial NO synthase (eNOS) and may compromise caveolar stability and the downstream signal transduction of caveolar proteins such as eNOS [30]. eNOS dysregulation, which reduces NO production, would favor increased oxidative stress, with the increased levels of ROS being central to the mechanism of Fabry’s cardiovascular disease. In fact, the α-GalA knockdown human endothelial cell line by RNA interference showed dramatically enhanced 3-nitrotyrosine (3NT) production in addition to Gb3 accumulation and reduced eNOS activity. The 3NT elevation is a result of a post-translational modification of proteins that occurs in situations of increased oxidative stress caused by reduced NO bioavailability [30]. Moreover, the excessive production of superoxide and other ROS reduces NO bioavailability via their chemical reaction with NO to produce peroxynitrites. This induces oxidative stress and cell damage via lipid peroxidation, as well as inactivates enzymes and other proteins by oxidation and nitration.

In erythrocytes of patients with Fabry disease that were undergoing ERT, decreased levels of antioxidant defenses were shown in terms of reduced glutathione and glutathione peroxidase activity as well as increased superoxide dismutase/catalase ratios [31]. The plasma level of malondialdehyde (MDA) and protein carbonyl groups were also increased in Fabry patients together with proinflammatory cytokines IL-6 and TNF-α. In addition, urinary Gb3 levels were positively correlated with IL-6, carbonyl groups, and MDA plasma levels [31], suggesting that proinflammatory and prooxidant states occur in Fabry disease, are correlated, and seem to be induced by Gb3.

In addition to the abovementioned evidence of oxidative stress activation in Fabry disease and given the close relationship between oxidative stress, oxidative stress signaling, and cardiovascular-renal remodeling, and the fact that studies from our laboratory have demonstrated using markers of oxidative stress and proteins related to oxidative stress such as p22^phox^ (a subunit of NADPH oxidase essential for the translocation of the electron to molecular oxygen to form superoxide) the triggering of the induction of free radicals and oxidative stress [32], lipoperoxides, and the RhoA/Rho kinase pathway [14,33], it appears likely that elevated proinflammatory cytokines, oxidative stress-related proteins, and oxidative stress signaling play important roles in the processes involved in the pathophysiology of organ damage in Fabry disease [6]. A better understanding of the biological and mechanistic processes that lead to endothelial dysfunction and disease progression in Fabry disease may suggest additive treatment strategies in order to lessen the high morbidity of Fabry patients.

We have recently provided clear evidence, using a molecular biology approach, that in Fabry patients under ERT, oxidative stress is activated in terms of the increased protein expression of p22^phox^ and increased level of MDA, suggesting that in Fabry patients oxidative damage affects lipid structures—contributing in the medium- to long-term to the onset of pathological processes and leading to cardiovascular-renal and cerebrovascular damage [34]. Furthermore, we have shown that the Rho kinase pathway is activated in Fabry patients, evidencing a significantly increased phosphorylation status of its target MYPT-1 and suggesting that Rho kinase activation may play an important role in the oxidative stress signaling and cardiovascular renal remodeling of Fabry patients [34]. In addition, we have also shown an altered reaction to oxidative stress in Fabry patients in terms of the reduced production of Heme Oxigenase-1 [34], induced by and protective toward oxidative stress [35]. This dysregulation—in addition to the Rho kinase signaling and oxidative stress activation—may further contribute to the cardiovascular-renal remodeling of Fabry patients, adding further information to our understanding of the pathophysiology of Fabry disease organ damage. This should stimulate further studies in order to gain further insights on the role of oxidative stress, oxidative stress signaling, and oxidative stress-induced inflammation in the cardiovascular-renal remodeling of Fabry disease. A better understanding of the specific molecular signaling responses to oxidative stress in Fabry disease could suggest additional interventions by either pharmacological or nutritional measures aimed at improving/halting the progress of cardiac and cerebrovascular disease and nephropathy that occur in Fabry patients. Moreover, this could lead to the improvement of their quality of life via the reduction of oxidative stress.

## 4. Conclusions

Fabry disease is a complex multisystemic disorder characterized by mostly nonspecific signs and symptoms. Given the importance and the impact of early initiation of a specific treatment to the disease course, efforts should be made to reduce late diagnoses.

ERT helps to slow the disease and alleviate symptoms but, in particular in the case of late diagnosis, has shown to be suboptimal because of cardio-cerebrovascular-renal outcomes and the occurrence of irreversible organ injury. There is thus the need for additional therapy to treat/slow/prevent the progression of the disease toward end organ damage. Evidence points toward oxidative stress as a factor involved in the pathophysiology of Fabry disease’s organ damage and the recognition that it may contribute to the medium- to long-term organ damage of Fabry patients might provide an opportunity for additional intervention. Studies are strongly needed to prove this chance on clinical grounds.
